# Investigating efficient urban planning strategies for reducing land surface temperature of the neighborhoods in Tehran, Iran

**DOI:** 10.1371/journal.pone.0313417

**Published:** 2025-04-21

**Authors:** Hadi Soltanifard, Abdolreza Kashki, Rahman Zandi, Majid Amani-Beni

**Affiliations:** 1 Department of Environment, Faculty of Geography and Environmental Sciences, Hakim Sabzevari University, Sabzevar, Islamic Republic of Iran; 2 Department of Geomorphology and climatology, Faculty of Geography and Environmental Sciences, Hakim Sabzevari University, Sabzevar, Iran; 3 Department of Physical Geography, Faculty of Geographical Sciences and Planning, University of Isfahan, Isfahan, Iran; 4 Department of Biomaterials, Saveetha Dental College and Hospitals, SIMATS, Saveetha University, Chennai, India; Sejong University, KOREA, REPUBLIC OF

## 1. Introduction

Nowadays, it has been widely highlighted that land surface temperature (LST) is an important indicator supplying scientific support to identify the urban heat island (UHI) phenomenon. LST can help experts assess the urban thermal behavior, internal climate among buildings, and flux energy exchange on a local scale through the continuous replacement of natural land cover with an artificial surface [1–5]. With continuously increasing concerns about rising temperatures in urban areas, broad scholars have paid attention to LST for a better understanding process, knowing the leading factors and mitigating adverse impacts and dimensions [6–11]. Although the research outputs have been widely addressed as the underlying forces on LST, the precise and comprehensive exploration of the parameters involved in urban planning and LST mitigation has remained a substantial scientific challenge.

Urban literature has revealed that urban components’ planning constantly seriously impacts urban climate and determines the degree of overheating [12,13]. In this regard, implementing measures to counteract or mitigate the heat effect depends on various factors, some of which can be incorporated into urban planning strategies [14]. Urban land use and activity, street geometry, urban trees and canopy types, morphology, building materials, height, and density are critical factors of planning indicators that urban planners have widely adopted to mitigate the intensity of the heat at the initial planning stage [15–20].

Alongside numerous studies, one of the most challenging issues in planning is limiting the obtained results to general strategies for reducing or replacing the effects of heat surfaces, which, in most cases, are the critical parameters in urban planning that have been ignored. In more cases, the lack of attention to scale and geographic location in explaining planning and management strategies must provide sufficient resolution to direct guidance for sustainable urban planning and development against heat emissions. Accordingly, the local governments must modify proposed guidelines to their specific circumstances, actions, and scale of practical interventions [21]. Hence, any insightful guideline should also consider the appropriate study scale for better and sustainable urban planning and development.

Concentrating on developed countries, however, there is a growing body of empirical studies to address LST alleviation and adaptation in big cities on a large scale, which has yet to be limited in demonstrating the implication of general strategies in urban planning. In contrast, by focusing on the potentials and constraints of approaches, possible strategies to build cooling cities for fast-growing and densely populated cities in developing countries like Iran have rarely been concerned [22–26]. Therefore, a systematic study that could bridge the gaps and establish possible mitigation strategies should also be considered.

This study’s main argument is to explore the effects of the leading urban indices on LST and heat emission in Tehran’s neighborhoods, Iran. Moreover, the study explores how urban planners can set up key strategies to combat the growth of heat surfaces according to the relevant outputs. This study was conducted in Tehran neighborhoods to better apply the detailed results in urban planning. The present study examined morphological indices, urban vegetation, and spatial patterns. Additionally, we used the space syntax method to quantify the spatial configuration of open space, which is a more elaborate way of looking at the spatial configuration of urban areas. Regarding space syntax, the spatial configuration implies the urban layout and existing relationships between the components of the built environment by providing a conceptual framework to describe the spatial arrangement of urban elements such as street patterns, blocks, and buildings [27,28].

The present study aims to address how urban planning strategies can effectively mitigate heat emissions by identifying the key factors specific to each studied region and context. The hypothesis is that comprehending the origin and characteristics of these contributing factors and their synergic impact will lead to the development of suitable strategies for reducing urban heat emissions. Notably, the present study has the following objectives: 1) Providing an analysis of the physical and spatial features of the urban neighborhoods and their impacts on LST; 2) Prioritizing urban indices through their proportion and impact on LST variations; 3) Extracting and classifying the urban neighborhoods as thoroughly influenced by the proposed components; 4) Proposing efficient strategies for the planning of neighborhoods based on the correlation and influence of the indices on LST changes. In recent decades, there have been numerous studies aiming to connect urban heat with urban planning strategies. However, the interaction between variables and their relationships at a small scale, such as in neighborhoods in arid and hot climates, is still a topic of debate. This study aims to address these gaps and present a logical framework to derive practical recommendations for urban planning. The results will give urban planners and managers more profound insights into impressive aspects of the urban indices on LST and adopt efficient strategies to alleviate LST effects on urban neighborhoods. In developing countries like Iran, it is essential to understand how to reduce the adverse effects of LST and provide guidelines for efficient urban planning to enhance the urban environment for dwellers by optimizing the local environment.

## 2. Data and method

### 2.1. Study area

**Fig 1:**
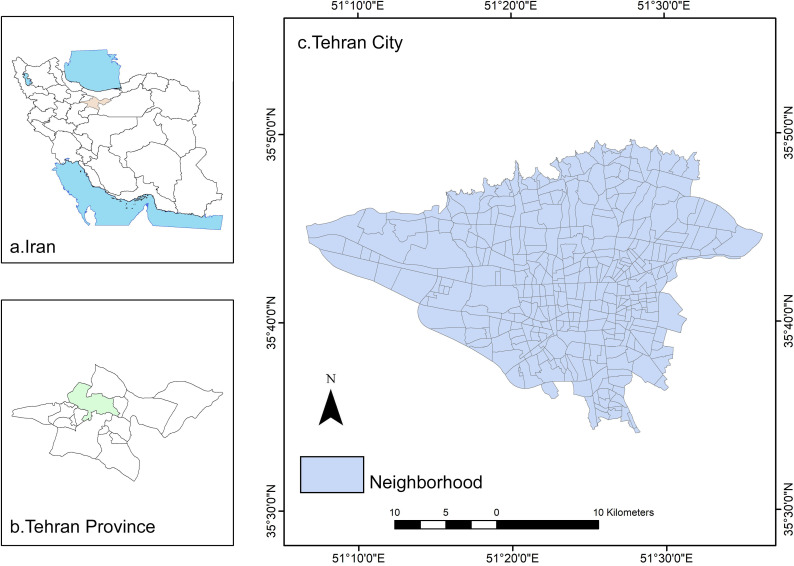
Location of the study area.

As the capital of Iran, Tehran is located relatively in the south of the Alborz mountains, within the range of 51°04′ to 51°47’ E longitudes and 35°31′ and 35°57’ N latitudes (Fig 1). Due to the city expansion of over 700 km2 and its geographic location, Tehran has mutable weather from the north and center (cooler) to the south (hotter). However, the study area annually has a long-term mean rainfall of 316 mm, 17°C temperature, 40% humidity, and 36 freezing days. Tehran has more than 8.7 million population, inhibiting 22 municipal districts and 367 neighborhoods. In addition, with 15 million in the large metropolitan area, Tehran is the most populated city in Iran and the third largest metropolis in the Middle East [29].

In recent decades, Tehran has been characterized by its rapid population growth, urbanization, and industrialization. Additionally, by increasing significant political, administrative, economic, and cultural roles, approximately 26% of all industrial units, 30% of the economy, and 40% of the consumer market in Iran are centralized in Tehran [30]. Hence, the city has historically experienced deep transformations in urban form and severe environmental problems such as land cover conversion, destruction of green areas, and the rise of LST. For instance, Tehran’s land cover has decreased from 72.19 km2 in 1988 to 30.14 km^2^ in 2018; in other words, 7.10% of the total land was converted into high-populated areas with great building density [31]. Furthermore, the city had a significant difference of nearly 3–9 °C of LST in Tehran rather than in the suburbs from 1988 to 2018 [31]. The LST progress directly affects the air quality, urban climate changes, and health of inhabitants in Tehran neighborhoods.

### 2.2. Data acquisition

This study utilized 20 Landsat 8 satellite images acquired between 2020 and 2021 to extract Land Surface Temperature (LST) and Normalized Difference Vegetation Index (NDVI) values. The imagery was downloaded from the United States Geological Survey’s (USGS) Earth Explorer online platform (https://earthobservatory.nasa.gov/). The specific details regarding the downloaded imagery, such as path, row, and date of acquisition, are provided in Table 1.

**Table 1:** Landsat 8 image specifications.

Number	Satellite	Sensor	Date	Spatial resolution
1	Landsat 8	OLI	14-Jan-20	Multispectral: 30 m Thermal band: 100m
2			30-Jan-20	
3			15-Feb-20	
4			2-Mar-20	
5			18-Mar-20	
6			3-Apr-20	
7			19-Apr-20	
8			5-May-20	
9			21-May-20	
10			6-Jun-20	
11			22-Jun-20	
12			8-Jul-20	
13			24-Jul-20	
14			9-Aug-20	
15			25-Aug-20	
16			10-Sep-20	
17			26-Sep-20	
18			12-Oct-20	
19			28-Oct-20	
20			15-Dec-20	

All satellite images were pre-processed and then processed in the Google Earth Engine by writing the script. Then, LST and the NDVI were calculated for all images.

The spatiotemporal resolution of the images was 30m for the multiple-spectral bands 1 to 7 and 9. In addition, thermal bands 10 and 11 with 100m resolution are suitable for retrieving accurate surface temperature. In our work, band ten was utilized to extract the heat data. This band provided more precise surface temperatures and was collected at 100 meters with wavelength 10.60–11.19 micrometers. As an image pre-processing step, atmospheric corrections were made by applying the FLLASH procedure before calculating LST to increase the accuracy of the LST output. However, after performing geometric image distortion correction, the Maximum Likelihood Classification (MLC) was the most widely used method with high precision and accuracy [32].

Moreover, other required data were obtained from the Tehran Master Plan in -2012 (https://rpc.tehran.ir/en/Publications/Books-Reports). The data, including urban morphology (building height and density, SVF), has often been presented at a block level. Urban road length and area are two independent variables from the -2012 Tehran Master Plan (TMP). Urban road data was also used to calculate the spatial configuration (integration Rn) for the entire neighborhood. Furthermore, urban vegetation and its spatial patterns (NDVI, Mean Proximity Index (MPI), and Mean Perimeter Fractal Dimension (MPFD)) were added to these sets of variables for each neighborhood. Tehran has 367 neighborhoods divided into 22 districts where the primary data were analyzed in each neighborhood. In this study, the average of variables in each neighborhood scale was generally applied; however, some variables, like the road network, are calculated as the sum of all the lengths of the roads in each neighborhood.

### 2.3. Variables

#### 2.3.1. LST data retrieval.

Several methodologies exist for extracting Land Surface Temperature (LST) from satellite imagery. This study employs a key analytical approach that involves converting the Digital Numbers (DN) of the Landsat thermal band into spectral radiance ($L_{\lambda }$ ). This conversion is achieved through (Equation 1) [33].


$$L_{\lambda }=\frac{\left(LMAX_{\lambda }-LMIN_{\lambda }\right)}{\left(Q_{Cal\max}-Q_{Cal\min}\right)}*(Q_{Cal}-Q_{Cal\min})+LMIN_{\lambda }$$ Equation 1

$L_{\lambda }$: spectral radiation

$Q_{Cal}$: numerical caliber DN

$Q_{Cal\min}$: Minimum of Q ^cal^ with LMIN_2_

$Q_{Cal\max}$: Maximum of Q ^cal^ with LMIN_2_

**Calibration Parameters:** To account for sensor characteristics, two calibration constants are introduced: LMIN and LMAX. These constants represent the minimum and maximum spectral radiance values corresponding to digital numbers (DN) of 0 and 255, respectively, within the **Landsat thermal band’s quantization range,** in $\left(wm^{-2}sr^{-1}\mu m^{-1}\right)$. The subsequent step leverages Planck’s equation (Equation *2*) to convert the spectral radiance values into temperature units [33].


$$T_{B}=\frac{k_{2}}{Ln(\frac{k_{1}}{L_{\lambda }}+1)}$$ Equation 2

In the Equation, T_B_ is brightness temperature (Kelvin) that represents the temperature of a blackbody that would emit the same radiance as the measured spectral radiance. $L_{\lambda }$ is spectral radiance $\left(wm^{-2}sr^{-1}\mu m^{-1}\right)$, and $k_{1}$ and are calibration coefficients in $\left(wm^{-2}sr^{-1}\mu m^{-1}\right)$.

The final step involves retrieving LST by incorporating an emissivity correction. Artis and Carnahan (1982) proposed the following equation (Equation 3) to account for atmospheric and emissivity effects during LST estimation [34]. The following Equation is:


$$LST=\frac{T_{B}}{1+(\lambda *\frac{T_{B}}{\alpha })Ln(\varepsilon )}$$ Equation 3

Where:

λ: Radiance emitted wavelength (11.5 μm)

C: the speed of light that is. $2.998*10^{8}(m/s)$

h: Planck’s constant value. $6/626*10^{-34}(j.\sec)$

$T_{B}$ = lightness temperature.

b: Boltzmann constant value $1.38*10^{-23}(j/k)$

α is equivalent to $\frac{hc}{b}=1.438*10^{-2}(mk)$

#### 2.3.2. Urban indices.

Given the rapid and unplanned development and the significant changes in population density and urban morphology over the past few decades, the study examined the factors affecting the LST rate in the study area. On this point, nine indices were chosen to describe the neighborhood features and their impacts on the LST. Our reasons for selecting all these indicators included their ordinary calculating, data availability, interpretation of standard physical, spatial, and natural aspects of neighborhood units, being easily understood by urban planners and policymaking, and having enormous referred resources with the participation of LST. Table 2 lists the applied variables.

**Table 2:** Urban form indicators selected in this study.

Category	Index	Description	Unit	range	Equation
Urban morphology	Building height(BH)	Expresses the height of the building	m	0<BH	–
	Building density(BD)	Shows by the proportion of building square footage in a block, neighborhood, or district. BD defines crowded or built-up a neighborhood, land intensity, and worth of land or building.	None	0≤BD	$\frac{Builduparea}{landarea}$
	Sky view factor(SVF)	Offers a more pertinent metric for characterizing urban geometry compared to methods that solely consider the ratio of radiation received by a surface to hemispherical radiation. SVF explicitly accounts for the proportion of the sky hemisphere visible from a specific point, providing a more comprehensive understanding of the radiative exchange processes within an urban environment.	Degree	0<SVF≤1	$2\pi -\overset{ND}{\underset{d}{\sum }}S_{Hd}$ Where ND is the total number of sectors discretizing the sky hemisphere; *d* is a specific azimuthal ray direction representing a particular viewing angle, and *SHd* is the surface area of the sky hemisphere segment obscured by obstacles in azimuthal direction *d.*
Urban road	Road length (RL)	This metric quantifies the total linear extent of roadways within a designated neighborhood boundary.	m	0<RL	$\overset{n}{\underset{i=1}{\sum }}L_{i}$ L_i_ is the length of the road in an urban neighborhood.
	Road area (RA)	This parameter represents the total surface area occupied by urban roads within a neighborhood.	m^2^	0<RA	$\overset{n}{\underset{i=1}{\sum }}L_{i}W_{i}$ Where *Li* is the length of the road, and *Wi* is the width of the road.
Spatial configuration	Integration-Rn (Integ_Rn)	It describes the average depth of a space to all other spaces. A value that is used to measure spatial configuration. Rn can represent integration patterns on a large scale and define small, close areas with geometric centers.	None	0<Int−Rn	$\frac{1}{RRA_{Rn}}$ RRA is a normalized measure since it is calculated as RA normalized through the D-value. Integration-Rn is the inverse of RRA [35].
Urban vegetation and spatial patterns	Normalized Difference Vegetation Index (NDVI)	It is a useful index to quantify vegetation greenness, density, changes, and health.	None	−1≤NDVI≤+1	The NDVI is conventionally computed as the ratio between reflectance values in the red (R) and near-infrared (NIR) bands of the electromagnetic spectrum. This equation is expressed as: $R_{NIR}-R_{RED}/R_{NIR}+R_{RED}$
	Mean proximity index (MPI)	It quantifies the isolation and fragmentation of urban vegetation. It provides a measure of the average size and separation of individual vegetated patches within the urban landscape.	None	0<MPI	$\overset{n}{\underset{i=1}{\sum }}\frac{a_{i}}{h_{ij}^{2}}$ In the equation, *n*, is the total number of vegetation patches within the analyzed urban landscape. *a*_*i*_ is the area of individual vegetation patch *i,* and *hij* is distance between the centroid (center) of patch *i* and the centroid of patch *j.*
	Mean Perimeter Fractal Dimension (MPFD)	It quantifies the complexity of the shape, with values ranging from 1 to 2. A value of 1 corresponds to patches with simple perimeters, such as squares, while a value approaching 2 indicates increasingly complex shapes.	None	*1 ≤ MPFD ≤ 2*	$\frac{\sum_{j=1}^{m}\left(\frac{2lnp_{ij}}{lna_{ij}}\right)}{n_{i}}$ Where *Pij* is perimeter of a patch belonging to type *ij,* measured in meters (m), *aij* is the area of a patch belonging to type *ij.* and N is the total number of patches belonging to a specific type.

As Table 2 briefly shows, building height (BH) is one of the most essential morphological factors to indicate LST in the urban environment. Buildings increase the exposed surfaces to sunlight radiations and trap heat in vertical space owing to their area and height [36]. Since this study was conducted at the neighborhood level, the average heights of buildings were employed. Moreover, building density (BD) is defined as the area, coverage, or footprint occupied by the building and is related to airflow, heat intensity, and ventilation conditions [37]. Additionally, the sky view factor (SVF) is the most appropriate parameter to represent the 3-dimensional form of the built environment where the sky is visible at a given location. Accordingly, SVF affects the spatial patterns of air and land temperature through street geometry and building density, changing the mean radiant temperature intensity [38,39]. Typically, it is defined as a dimensionless measure ranging from 0 to 1, implying solar radiation intensity and representing completely obstructed and free space, respectively [40]. There are various approaches to calculating SVF by different sets of data; however, in the current work, ground, building, and vegetation digital surface models (DSM) were used to generate SVF by the SVF plugin in QGIS 3.4 [41].

Urban roads and networks are also prominent urban indices that affect thermal characteristics in this study [42]. The current work has focused on relative changes in LST concerning the characteristics of urban roads, such as the road length and area at the neighborhood level. These metrics provide a better basis to compare the performance of urban roads among the neighborhoods about LST. Moreover, to better understand the role of urban road configuration in LST difference, the global integration Rn, which is widely applied based on street network analysis, was chosen. In summary, Integration Rn means global integration, spatially measuring the value of an accessible street by the distance or depth of all other city streets [35]. Indeed, Rn symbolizes global integration and is computed using the unconstrained radius (from any space to any other in the system). Ultimately, ArcGIS 10.3’s zonal statistics tool determined the values within each neighborhood after calculating the street network using the axial line approach in the UCL depth map 10.

Urban vegetation, or the NDVI, is a domain factor in LST derivation. Fundamentally, spatial patterns of LST have been proven to be associated with land cover in the urban environment. Hence, the NDVI is a significant factor in detecting the temperature of surface emissivity in urban areas. Furthermore, to analyze spatial patterns of urban green patches, we employed landscape metrics to investigate the degree of fragmentation and complexity of existing green patches of Tehran’s neighborhoods. In this study, the degree of isolation and fragmentation of urban vegetation is quantified by the Mean Proximity Index (MPI). MPI refers to the connectivity of green space patches, which increases with a higher MPI value [43,44]. As the neighborhood becomes more occupied by patches of the same type and as those patches become closer, more connected, and less fragmented, MPI increases. In addition, the Mean Perimeter Fractal Dimension (MPFD) was chosen as a landscape metric to assess the complexity of the green shapes. MPFD is derived from the perimeter and area of a patch, and it characterizes the intricacy of the patch. The Coefficient of Variation is standardized based on the mean value and can be compared across different landscapes [45,46].

### 2.4. Method

In summary, the following steps were taken to implement the study:

1) The key variables were selected to measure the value of all driving factors.
2) The raw values were converted to the standard values to obtain the comparable factor from different sources.
3) An association between the intensity of LST and variables was investigated by carrying out the Geographic Weight Regression (GWR), Ordinary Least Squares (OLS), and spatial autocorrelation (Moran I) values to compare results.
4) The warmest and coolest neighborhoods were classified into two groups, and then the t-test was performed to detect influencing factors.
5) Finally, effective strategies against heat risk were proposed at the neighborhood scale. The t-test was performed to detect heat risk at the neighborhood scale.

## 3. Results

### 3.1. LST spatial patterns

**Fig 2:**
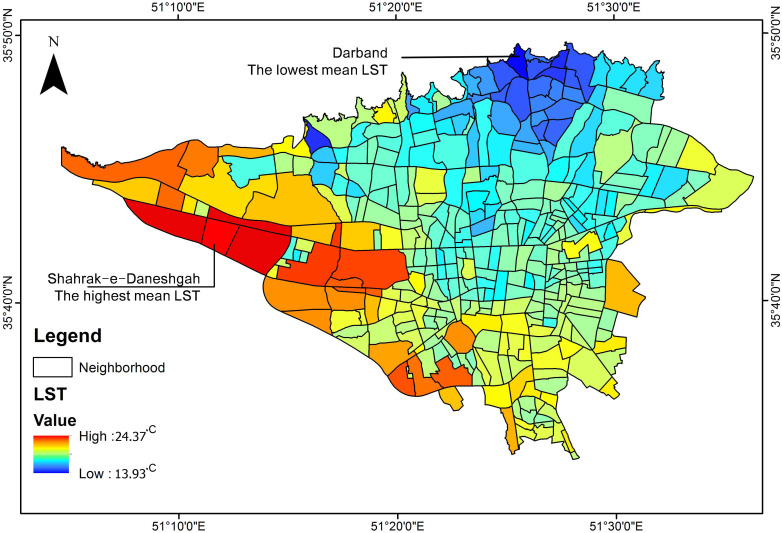
Calculated mean LST in Tehran urban area. In the heat map, red shows the hottest urban surfaces, and blue represents the coldest urban areas.

The average LST was estimated based on the original surface temperature to reasonably compare the neighborhoods. As Fig 2 illustrates, LST gradually increased from the north to the south and the east to the west of Tehran. Specifically, the lower LST value was primarily concentrated in northern neighborhoods, and higher values of LST were scattered in the neighborhoods in the west and south of the study area. Accordingly, the highest average LST extracted in the neighborhood of Shahrak-e-Daneshgah was 24.37°C, and the lowest average of LST observed in Darband neighborhood was 13.93 °C.

Although Shahrak-e-Daneshgah has recorded the highest mean LST in this study, the results reported that most of the bare and industrial lands experienced LST in the range of 22-24°C, which were concentrated in the west and southwestern of Tehran or located within the urban neighborhoods such as Mehrabad airport, Esteghlal, and Tehransar. In contrast, the northern neighborhoods such as Baharan (northwestern part of Tehran), Dezashib (northern part of the city), and Zaaferanieh experienced LST in the range of 13-14 °C. This temperature difference has been mainly observed due to the vegetation loss and growth of impervious surfaces, land use type and current activities, green spaces, and existing water bodies in the studied areas.

### 3.2. Spatial distribution of the variables

**Fig 3:**
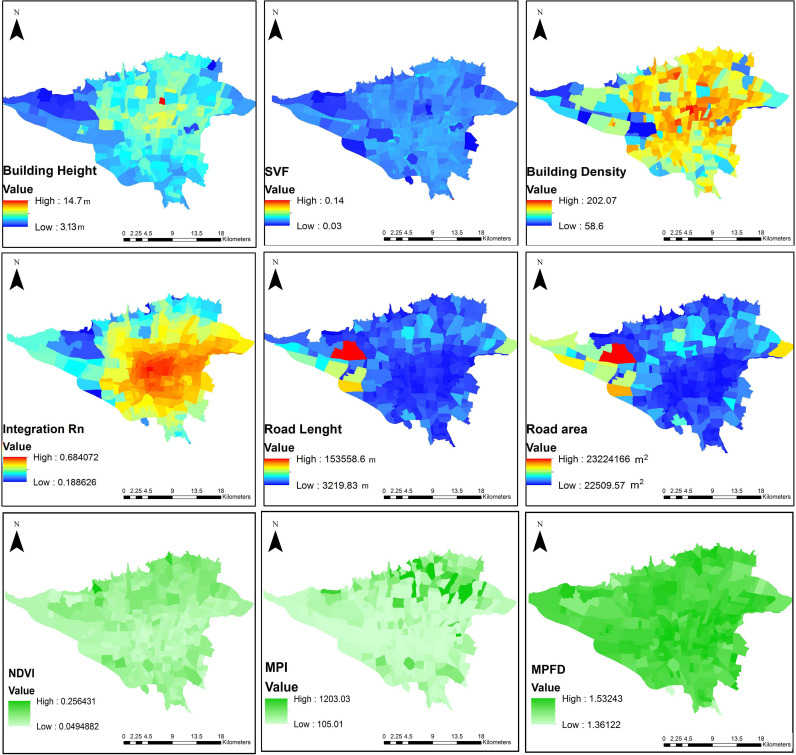
Spatial distribution of the selected variables.

The selected variables presented different spatial distributions (Fig 3). Building height (BH), building density (BD), and integration Rn (Int_Rn) displayed relative changes from the city center to the suburbs. Contrary to road length (RL), road area (RA), NDVI, and MPI, the variables’ values were typically high in the center and became low toward the edge. However, no regularity was seen in the spatial pattern of MPFD and SVF.

### 3.3. Regression models

**Fig 4:**
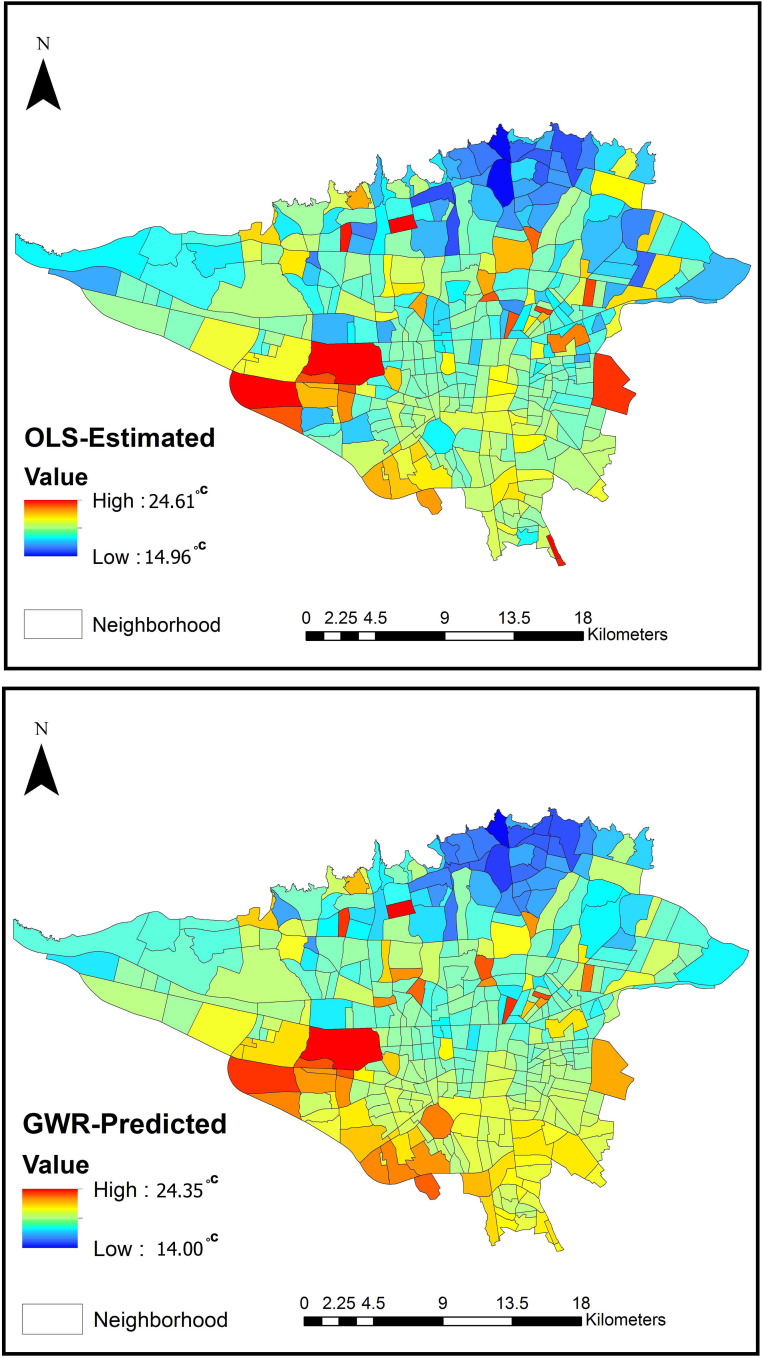
The predicted value of LST in the study area.

The OLS and GWR models were conducted to explore how the characteristics of the variables led to LST variations in our work. LST was considered a dependent variable, while others were considered independent variables. Furthermore, the Global Moran’s I tool was employed to measure spatial autocorrelation based on feature locations and values. Fig 4 indicates the predicted LST values and their distribution across the study areas for both models.

The OLS results illustrated that BD, NDVI, BH, SVF, and MPI had significant negative impacts on LST, where among all the negative indices, BD had the most considerable influence on LST. In contrast, RA, MPFD, Integ_Rn, and RL were positively correlated with LST. Although, among all the confirmatory parameters, RA had the most significant effect on LST. However, the effect size was observed with low R-squared levels. The final regression equation appears as follows.


$$LST=40.567-0.909\left(BD\right)-0.791\left(NDVI\right)+0.333\left(RA\right)-0.427\left(BH\right)-0.336\left(SVF\right)-0.235\left(MPI\right)+0.236\left(MPFD\right)+0.295\left(Integ\_Rn\right)+0.288\left(RL\right)$$

The coefficient of determination (R^2^) is 0.629; therefore, the model shows that the predictors explain 62.9% of the LST spatial pattern with the *p-*value = 0.000 for all variables, indicating high significance. Compared to OLS, the GWR results represented an improvement in R^2^ value (0.803) rather than the OLS model, while the AICc was significantly decreased (Table 3).

**Table 3:** Fit metrics for OLS and GWR.

Variable	OLS	GWR
**R** ^**2**^	0.629	0.803
**AICc**	1164.14	1029.16
**Moran’s I**	0.167	0.023
	Pvalue: 0.000^*^	Pvalue: 0.000^*^

* p-value >  0.05.

**Fig 5:**
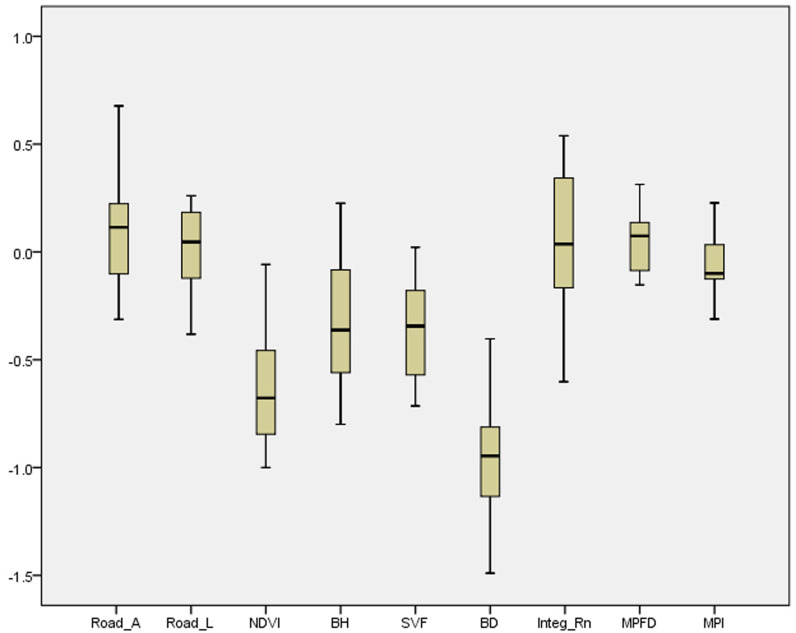
The box plot of the coefficient of variables.

Considering the significance of Moran’s I for the models and validating the spatial clustering, the obtained values indicate that GWR is suitable for interpreting the intensity of the variables. Therefore, GWR is a more accurate predictive model that reflects the relationship between the LST and the explanatory index of each neighborhood due to local spatial non-stationarity. Fig 5 displays the statistical distribution of the coefficient for the group variables. The box plot indicates that BD is still essential in reducing LST. With a negative coefficient, NDVI, BH, and SVF are other crucial factors on LST at the neighborhood scale.

As the most robust predictor, BD showed the highest decreasing trend in the mentioned equation, where a 1-point increase is associated with a 0.9-point decrease in LST changes. For the morphological aspect in the study areas, the BD (-66.8%), BH (-42%), and SVF (-37.3%) aggregately exhibited a negative effect on LST. In other words, building density and height demonstrated a significant negative contribution in descending LST owing to the heterogeneous architectural layout that impacts local temperature through air ventilation. In this study, the NDVI is a primary factor in alleviating LST, while the coefficient of determination (R^2^) is -0.607. Considering the dark surface of roads and higher heat absorption, urban networks played the highest role in LST changes. Based on the results, the road area(RA) and length (RL) were identified as the main contributors to increased LST by extending impervious surfaces with low albedo. Accordingly, a 1-point growth in RA and RL is directly related to a 0.33- and 0.188-point increase in the LST value, respectively. The road area, however, is a variable that has the strongest positive correlation (0.372) with LST on the neighborhood level, while the road length had the lowest impact (0.118). Compared to road length, urban network configuration (Integ_Rn) characteristics had a little more positive effect on LST (0.121), indicating the impact of the neighborhoods’ spatial structure on intensifying heat issues. Finally, two landscape metrics, MPI and MPFD, displayed a significant relationship with LST. Consequently, MPI had a negligible reducing effect on LST (−0.121), while a green patch’s average degree of isolation and fragmentation was measured at its minimum size. Although MPFD describes the shape complexity of the green patches, the average MPFD positively affected LST (0.128) due to the structural instability, spatial configuration, and intensive human influence on green patches.

### 3.4. Statistical analysis

Two groups of neighborhoods with the coldest and warmest temperatures based on the GWR predicted model were selected to identify the main contributors and better understand their proportion on LST. To choose two studied groups logically, 25% of the neighborhoods with the highest temperature compared to the mean LST were picked out in the warm group. The same proportion was also considered for choosing the neighborhoods that had the lowest average temperature compared to the average LST in the cold group. The selected neighborhoods include 76 samples (36 cold and 40 warm neighborhoods), classified in the groups. Table 4 presents the statistical characteristics of the variable for two groups.

**Table 4:** Statistical characteristics of the studied indices in the neighborhoods with cold (1) and warm (2) LST.

Variable	Group	Mean	Std. Deviation
**BH**	1.00	0.3721	0.90116
	2.00	-0.7920	0.89372
**BD**	1.00	0.4104	0.69529
	2.00	-0.9682	1.04526
**SVF**	1.00	0.1881	0.72094
	2.00	-0.5775	1.43158
**RA**	1.00	-0.1503	0.63883
	2.00	0.1677	1.24368
**RL**	1.00	-0.0910	0.59745
	2.00	0.1089	1.27641
**Integ_Rn**	1.00	-0.5375	1.09038
	2.00	-0.3565	0.89287
**NDVI**	1.00	0.8316	1.27267
	2.00	0.1415	0.87247
**MPI**	1.00	0.7094	1.44891
	2.00	-0.0332	0.93967
**MPFD**	1.00	0.1583	0.91104
	2.00	0.3506	0.90102
**LST**	1.00	15.6868	1.29095
	2.00	22.7188	1.66974

Then, a t-test was employed for the independent samples to compare the average of two samples and determine whether the mean values of the two separate groups differ significantly. The t-test is a parametric test to compare the mean and variance of two independent statistically distinct groups measured on an interval scale. A significant difference was hypothesized regarding the selected variables between the coldest neighborhoods with code 1 and the warmest neighborhoods with code 2. Table 5 shows the results of the analysis.

**Table 5:** Independent t-test results to compare cold and warm neighborhoods in terms of the selected indices.

Indices	t-test for Equality of Means		
	t	Sig. (2-tailed)	Mean Difference
**BH**	7.366	0.000	1.716
**BD**	8.806	0.000	58.459
**SVF**	3.827	0.000	0.004
**RA**	-1.822	0.031	95.900
**RL**	-1.136	0.028	91.070
**Integ_Rn**	-1.032	0.0304	-0.020
**MPI**	3.459	0.001	129.451
**MPFD**	-1.206	0.023	-0.002
**NDVI**	3.597	0.000	0.021
**LST**	-16.603	0.000	-7.032

Overall, there is also a significant difference between the two groups regarding the LST average. The T-Test results showed significant differences in other measures between the two groups; in a two-sided test (Two-way), the confidence level of 95% of the sig value is less than the 0.05 level. As a result, the average LST in group 2 is 7.032°C warmer than in cold neighborhoods, which is a significant average difference. In total, the mean LST in the cold neighborhoods is 15.68°C, and in the warm neighborhoods, it is 22.71°C. The average values of BH, BD, NDVI, SVF, and MPI in the group with cooler LST are higher than in areas with warmer ones; hence, the average difference of these five indices is significant in the groups.

## 4. Discussion

### 4.1. Factors and spatial analysis

**Fig 6:**
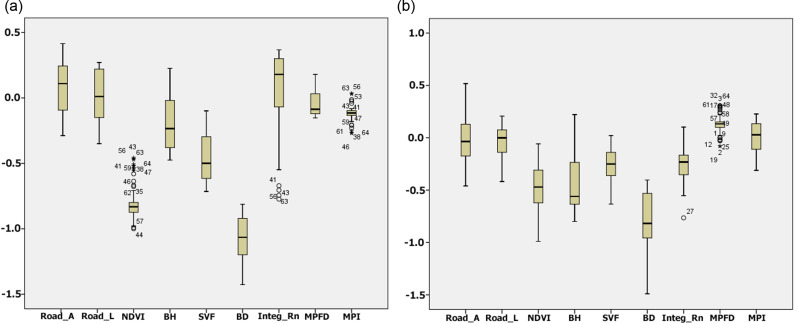
Box plot of the variables in the studied groups (a: the cold group and b: the warm group).

The research findings indicate that GWR had a better performance than OLS. However, one of the main arguments of the study was to identify the local details of the variables that affected spatial variation in LST and help us effectively plan urban neighborhoods against heat. By comparing the two study groups (Fig 6), it was observed that BD had a notable cooling impact on LST. Accordingly, the average density of buildings in the cold group was recorded as 138.26, whereas it was measured as 97.911 in the warm group.

Overall, the relationship between building density and LST has rarely been understood in such studies without examining climatic zone conditions [17]. However, few previous studies have reported a significant growth in LST value along with increasing building density [37,38,47,48], whereas several studies have confirmed that urban areas with high density of buildings had a negative association with LST, particularly in arid and semi-arid areas [49–53].

Similarly, this phenomenon was also observed between the height of buildings and LST variation in the study neighborhoods. Based on the results, BH had a significant positive cooling effect on LST. Specifically, the results recorded that the average BH in the cold group equals 7,612 m, whereas in the warm group was 3,281 m. In other words, the neighborhoods with high-rise buildings showed lower average LST than those with mid and low-rise buildings due to large surface areas covered by buildings’ shadows. However, the relationship between BD and LST variation is complex and constantly depends on the geographic location of the study area. However, several scholars have emphasized the effect of the height of buildings on impeding air movement and hindering the exchange of cooled air in an urban environment [54–58], a growing body of research mainly in hot- arid climate has represented the cooling effect of taller buildings through shading a large area and shielding more solar radiation [52,59–65]. As well as other morphological aspects in the neighborhood, SVF showed a negative correlation with LST variation. In the two study groups, it was found that the amount of SVF had a significant difference, with the average value of SVF in the cold group being 0.0544 and the same in the warm group equaling 0.0488. Accordingly, it might be concluded that the more significant SVF value is concentrated in the neighborhoods generally surrounded by higher building density and taller buildings [39,52]. These morphological features, as well as vegetation, provide a continuous cooling effect by shading large urban surfaces and creating obstacles to the absorption of solar heat waves [66–70].

Among all the factors considered in this study, it was observed that the NDVI had relatively the most significant negative effect on LST change after building density. However, the negligible difference between mean NDVI in the selected groups (0.052) indicated that the cooling effect of urban vegetation might be a synergetic outcome between the morphological properties, NDVI, and spatial patterns of green patches, leading to low LST. In other words, in neighborhoods with mid- and low-rise buildings, the NDVI performed better than the cold group in alleviating the heat [71]. To confirm this claim, investigating the spatial patterns of urban vegetation in two groups (MPI and MPFD) showed significant differences (1.3 and 0.168), respectively, meaning that a large amount of fragmentation and the mean complexity level of the urban green patches were main contributors in the cold group, which led to extending spatial heterogeneity and hence, very trivial effect on LST [72]. The neighborhoods with the lowest LST had relatively rich urban vegetation and more fragmentation and spatial heterogeneity in urban green space due to rapid urbanization. On the contrary, in the warm group, the cooling effect of urban vegetation showed the highest spatial variation in the NDVI values, which had a more significant negative impact on LST.

The characteristics of urban roads and their spatial configuration were other main contributors to predicting LST values in our work. Urban roads may primarily cause increasing daytime heat due to pavement, traffic, configuration, and geometry [73]. Fig 6 showed that RA, RL, and Integ_Rn all resulted in a positive effect but a small-fluctuated impact on LST variation; nevertheless, the results demonstrated a significant difference in the quantity and direction of the variables on LST values in the analyzed groups. The most essential characteristics of urban roads, which refer to the highest level of urbanization, are expected to increase surface temperature and lead to a warming environment [74]. Therefore, the sum of road length and area values in each neighborhood are the main drivers leading to heat.

Moreover, the general cover of urban roads and streets is asphalt pavement, which absorbs, stores, and releases more heat than natural surfaces. Hence, the findings demonstrated that RA and RL, particularly in RA, had a higher variation range in the warm group, leading to a warmer neighborhood. Besides these, the spatial configuration of the urban roads has also caused intensifying heat emissions in the studied neighborhoods. In this regard, the global integration (Integ_Rn) of the streets, roads, and pathways in the warm group demonstrated a considerable average (0.483), while the same size was 0.377 in the cold group. The integrated urban network implies highly accessible streets formed by linked surfaces across the city; therefore, an increase in the Integ_Rn level might result in extended heat surfaces and waves. As shown, the overall structure of neighborhoods is constructed by linking short, complicated, and indirect lines, whereas most neighborhoods in the opposite category are planned and developed with long, direct, and comprehensive access lines.

Furthermore, these streets, roads, and pathways, with relatively lower Integ_Rn, are surrounded by high-rise buildings that constantly shade the urban streets and provide a cooling effect during the days, whereas in the warm group, mid and low-rise buildings cannot create such circumstances. In the last decade, the concept of configuration and its influence on heat concerns has been focused on landscape elements. Therefore, urban configuration and its driving factors have received less attention [75–78].

### 4.2. Proposed strategies for neighborhood planning

Since Tehran is situated in the central part of Iran, its climate is influenced by its geographic location, where the Alborz mountain is located on its north, and the central desert of Iran is on its south. Tehran’s climate is, therefore, commonly characterized by its semi-arid characteristics, showcasing predominantly warm and sunny summers as well as cold and rainy winters. In addition to the microclimate characteristics of Tehran, the extent of the neighborhood units and the diverse features of the built environment makes it more necessary to develop effective strategies to deal with the increased urban heat. Therefore, this section provides long-term strategies crucial for both the natural and built environment to assist experts in planning the studied neighborhoods in the face of rising temperatures. These strategies are included as follows.

#### 4.2.1. Conserving existing gardens and existing urban vegetation.

**Fig 7:**
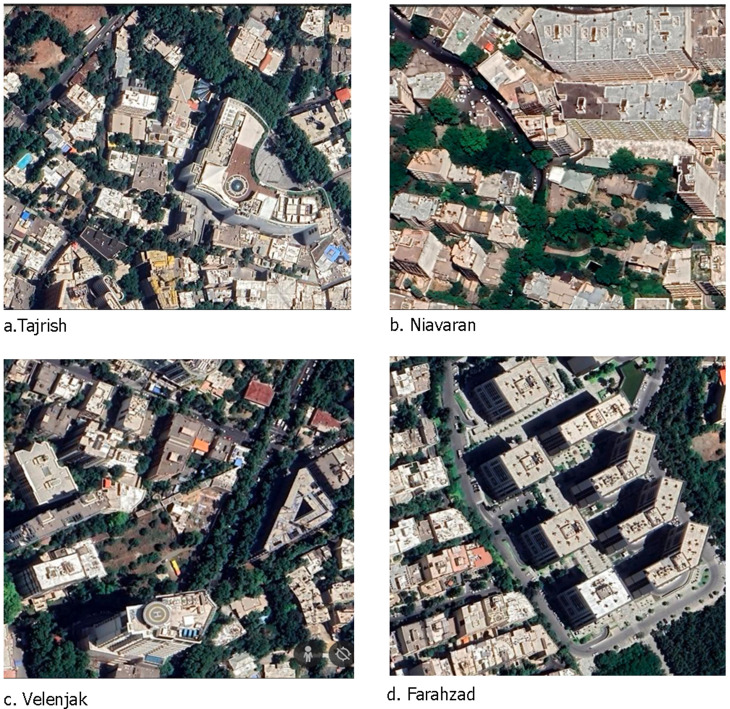
Some samples of the integration between high-rise buildings and urban green space for mitigation LST in the coldest neighborhoods. Source: Imagery ©2024 Airbus, CNES/ Airbus, Maxar Technologies Map data ©2024.

According to the results, the highest NDVI value (0.25) was observed in the northern and north-eastern parts of the city due to the presence of dense gardens and vegetation, whereas the lowest NDVI (0.04) has been recorded on the southern and south-eastern sides of the city due to the scattered vegetation and fragmented urban green space. The rapid increase in population growth over the past three decades and the extensive need for land for housing development have been the primary factors contributing to the removal of urban vegetation. This removal has led to the significant expansion of impermeable surfaces and increased LST [31]. Although there has been a rise in urban green spaces in the previous decade, the transformation has predominantly occurred in the neighborhoods in the northern part of the city. Specifically, Chizar, Jamaran, Dezashib, Niavaran, Tajrish, Elahiyeh, Velenjak, Zaafarnieh, Shahran, Ozgol, Sa’adat Abad, and Farahzad are the neighborhoods that have experienced such changes, which were previously home to the old and extensive gardens of Tehran (Fig 7).

In addition to considering the density of buildings, the preservation of gardens and other forms of urban vegetation can be a highly effective strategy for mitigating the LST effect in these neighborhoods. This approach reduces the LST effect and offers numerous benefits through positive synergy with high-rise buildings and creating shading effects.

#### 4.2.2. Ecological planning and urban green infrastructure development.

Although the results revealed that the morphological factors played a greater role in reducing the size of LST, it is essential to integrate urban planning systematically into urban green space planning to achieve more effective approaches to combat heat emissions. In this regard, ecological planning can be considered a nature-based solution approach to improving the distribution, connectivity, homogeneity, and spatial patterns of urban green space at the neighborhood scale. Accordingly, urban green infrastructure can be a viable solution to mitigate heat intensity in the studied areas. This approach can integrate urban-ecological planning and promote ecosystem services of urban green spaces to be more resilient and reduce the effects of LST. Implementing urban green infrastructure involves strategically placing natural and semi-natural areas throughout neighborhoods alongside other environmental features, thereby creating an interconnected green network that effectively reduces LST.

#### 4.2.3. Densification and planning of high-rise building.

**Fig 8:**
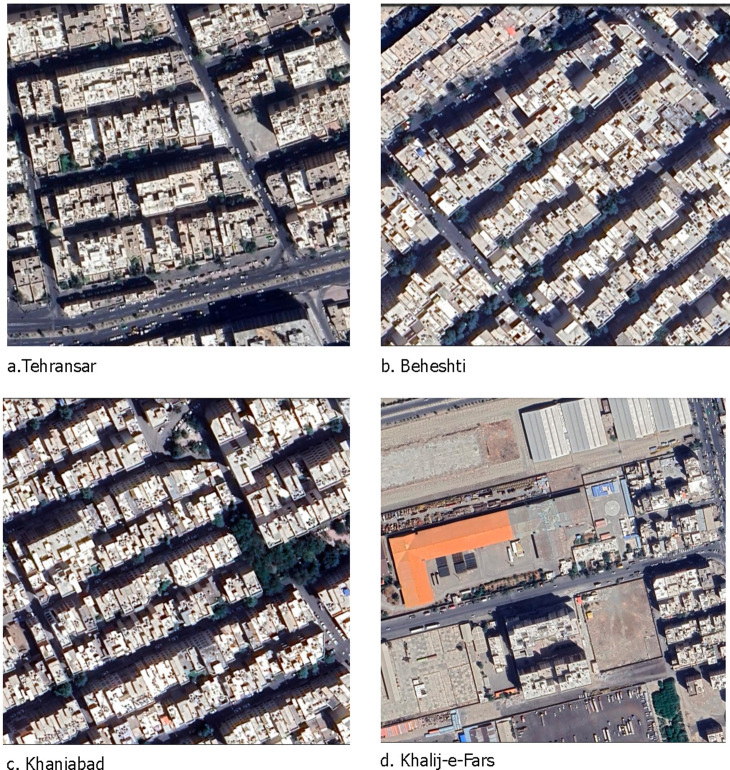
The examples of the urban morphology and lack of urban green space in the warmest neighborhoods. © 2024 by Imagery ©2024 Airbus, CNES/ Airbus, Maxar Technologies Map data ©2024 is licensed under CC BY 4.0.

Overall, the findings demonstrated a notable difference in the cause of the increase in the size of the LST level in the southern and western neighborhoods compared to the northern neighborhoods in Tehran. In recent years, there has been an increase in land expansion, a decline in urban green space, and the construction of smaller residential units with moderate to low height in the southern and western parts of Tehran. Specifically, neighborhoods such as Nemat Abad, Ismaeel Abad, Resalat, Beheshti, Khani Abad on the south side, and Khalij-e-Fars, Golshahr, Froudgah, Tehransar, Shahrak-e- Esteghlal, and Fath on the western side have experienced these changes (Fig 8).

These transformations can be attributed to the significant population growth and the resulting boost in demand for housing and relevant vacant lands. Therefore, it is apparent that these massive transformations have already enlarged the LST level. Since the southern and western neighborhoods displayed higher levels of variation in LST due to their urban morphology, characterized by medium building height and lower density, urban planning can effectively address this issue by adopting a strategy combining high-rise and high-density building clusters. Additionally, combining existing urban green spaces can reduce LST, making it a valuable approach.

### 4.3. Limitations and future aspects

The study identified several limitations that need to be addressed in future research. These limitations are as follows:

1. **Data limitations:** Difficulty in accessing current data, such as recent building density information, for model simulations and initial calculations.
2. **Monitoring challenges:** Difficulties in monitoring changes at the neighborhood level due to limitations in data availability and collection methods.
3. **Lack of a central system:** Lack of a centralized system for monitoring urban heat issues, including emissions sources and types.

Despite these limitations, the study emphasizes the crucial role of urban vegetation in reducing urban heat, especially in hot and arid climates. Future research should concentrate on investigating the types, composition, and arrangement of urban plants to enhance their cooling effectiveness.

## 5. Conclusion

The present study demonstrated that the morphological characteristics had the most significant negative impact on the intensity of LST in the neighborhoods of Tehran by comparing the investigated indices. However, this study presented a systematic assessment in three steps. Firstly, the research models, GWR and OLS, were utilized to examine the spatial distribution of LST, the variables on the study scale, and the relationship between them. Secondly, the key factors were indicated by assessing significant differences between the detected groups, where the causes of increasing LST in the groups were quite distinct. Lastly, based on the results, the study proposed appropriate planning strategies to address the variation in LST at the neighborhood level based on high-rise buildings and density and UGS optimization instead of new residential and UGS development. However, this study aimed to evaluate the most impactful variables despite the inevitable constraints in data gathering because of the limited data sources. Furthermore, considering the challenge of applying results from other cities with diverse climates, the present research also explored fundamental strategies that emerged from the findings. These strategies can be considered a valid source of mitigating urban heat in arid and hot climates, where case studies have rarely been discussed.
